# Detection of acute coronary occlusion with a novel mobile electrocardiogram device: a pilot study: reply

**DOI:** 10.1093/ehjdh/ztae043

**Published:** 2024-09-18

**Authors:** George Thornton Ratcliffe, William John Wallace, Gishani Poopalasingam

**Affiliations:** Lewisham and Greenwich NHS Trust, UK; Lewisham and Greenwich NHS Trust, UK; Lewisham and Greenwich NHS Trust, UK

We thoroughly enjoyed reading the manuscript ‘Detection of acute coronary occlusion with a novel mobile electrocardiogram device: a pilot study’ by Zepeda-Echavarria *et al*.^1^ The authors of this pilot study provide innovative insight into the potential of implementing a mobile handheld device that can record a multi-lead electrocardiogram—the miniECG. This manuscript provides a preliminary assessment into the capacity of this device in triaging patients with cardiac chest pain to simultaneously improve ‘symptom-to-door’ time and clinical prioritization for urgent coronary intervention. Within the National Health Service (NHS) in the UK, the scope for identifying appropriate triaging techniques and streamlining pathways to increase efficiency is at an all-time high. However, a delicate and comprehensive assessment is required for such co-morbid presentations such as acute coronary syndrome (ACS) and typical and atypical chest pain.

During March 2024, 1 433 300 patients attended NHS emergency departments with 15% of these presentations coded as a cardiac presentation.^2^ These numbers highlight the significant financial and workforce burden added to our emergency departments by chest pain attendances and admissions. If an alternative device were to be implemented in the triaging of chest pain patients in primary care, an important determining variable of its feasibility would be the specificity of the device in order to reduce the number of avoidable chest pain attendances to NHS emergency departments. The positive effects of e-health devices on hospital admission rates have already been evidenced in the UK’s National Health Service. Appropriate implementation of remote health monitoring through smart devices has led to a reduction in care home resident emergency admissions by ∼25%.^3^ This is just one example highlighting the potential for streamlined digital health triaging in alleviating the strain on NHS emergency services.

Women have been shown to present with higher rates of ‘atypical symptoms’ including abdominal discomfort and shortness of breath.^4^ In addition to this, studies show that women presenting to A&E with symptoms suggestive of ACS are triaged less effectively and are more likely to be discharged than men. Ultimately, the above reasons contribute to why women presenting with ACS have a significantly higher hospital mortality rate than men.^4^ Zepeda-Echavarria *et al*. noted that a higher proportion of females compared to males had ST-segment elevation missed on the miniECG that were then found to be present on a 12-lead ECG. This was suggested to be secondary to excessive chest wall adipose tissue.^1^ Ultimately, these findings imply that the miniECG could further compound the already present health inequalities that exist in ACS diagnosis and prognosis between genders. The concern lies that if the miniECG were to be used in chest pain triaging and identifying which patients require urgent primary coronary intervention, it could further worsen gender health inequalities in ACS and widen the prognostic gap. In order for future developments regarding the investigation and management of ACS to positively affect the patient demographic, there needs to be significant emphasis into the specific outcome it has on women.

We believe that there could be scope of implementing the miniECG in triaging patients who are already hospital bound, prior to a definitive 12-lead ECG to allow further diagnostic work-up. However, the authors of the paper also reports that the miniECG could be used to detect ST deviations in patients presenting with intermittent or atypical chest pain symptoms. The paper proposes that in this patient demographic, a miniECG could be used detect ischaemic changes that would warrant rapid referral. Our concern with this suggestion is that studies have shown that chest pain severity and persistence have been a poor predictive tool in ST-segment elevation diagnosis.^5^ With the miniECG recording a sensitivity of 65% for detection of occlusive myocardial infarction (OMI), it would be clinically irresponsible to deny patients who present in the community with atypical and none-persistent symptoms a formal 12-lead ECG and triage solely via the miniECG. In time-dependent conditions such as ACS, a sensitivity of 65% is insufficient for a test to be used in isolation to rule out a diagnosis.

We agree that there is a market for identifying and streamlining patients who require coronary intervention and further rapid diagnostic work-up and treatment through the use of easy access novel ECG devices. Our main concern lies with the prospect of the miniECG being used in the community to identify patients that require rapid referral. In the results section of the paper, it is reported that 21.4% of the patients were considered poor quality for interpretation in the context of ischaemia and excluded. Given that the interpretation was performed by cardiologists, this high proportion of one in five readings being poor quality raises the question of who would be interpreting the results of these miniECGs. In the community, these low-quality recordings wouldn’t have access to a formally trained electrophysiologist to determine whether these patients justify an alternative investigation (such as a formal 12-lead ECG) as it would be unknown if the person is having an OMI or not; thus, increasing the burden on healthcare services and potentially jeopardizing patient care and safety.

Overall, this innovative and thought-provoking study shows us the future capabilities of diagnostic tools in healthcare. We believe that at present, this study has demonstrated that there are some limitations with this device and thus need for further improvement. This is especially within the female demographic; an at-risk population with regard to ACS management and prognostic outcomes in which there is a large potential for improving such health inequalities through digital healthcare.

## Data Availability

No new data were generated or analysed in support of this research.
